# Did the COVID-19 Pandemic Change the Clinical Presentation and Management of Paediatric IgA Vasculitis? A Multicentre Retrospective Cohort Study

**DOI:** 10.3390/jcm15218399

**Published:** 2026-10-28

**Authors:** Maria Koźba-Baranowska, Małgorzata Stańczyk, Aleksandra Komorowska, Julia Kaliska-Midera, Agnieszka Wosiak, Elżbieta Smolewska, Agnieszka Brzozowska, Marcin Tkaczyk

**Affiliations:** 1 Department of Paediatrics, Immunology and Nephrology, Polish Mother’s Memorial Hospital Research Institute, 93-338 Lodz, Poland; maria.kozba-baranowska@iczmp.edu.pl (M.K.-B.);; 2 Department of Paediatrics, Immunology and Nephrology, Medical University of Lodz, 90-151 Lodz, Poland; 3 Department of Paediatric Cardiology and Rheumatology, Maria Konopnicka University Paediatric Centre, 91-738 Lodz, Poland; 4 Department of Allergology and Paediatric Internal Medicine, Dr Janusz Korczak Paediatric Centre, 92-328 Lodz, Poland; 5 Faculty of Technical Physics, Information Technology and Applied Mathematics, Lodz University of Technology, 93-005 Lodz, Poland

**Keywords:** IgA vasculitis, IgAV, COVID-19 pandemic, paediatrics

## Abstract

**Objectives**: Immunoglobulin A vasculitis (IgAV) is the most common systemic vasculitis in children and is frequently preceded by a respiratory or gastrointestinal infection. The COVID-19 pandemic affected the epidemiology of infections across multiple paediatric conditions and was accompanied by changes in hospitalization criteria, therapeutic practice, and healthcare organization. The aim of this study was to compare the clinical presentation and treatment of IgAV in children before and after the onset of the COVID-19 pandemic. **Methods**: We retrospectively analysed the medical records of 160 patients aged 0–18 years who were hospitalized for IgAV between 2015 and 2023 at three paediatric centres in the Lodz region. Patients were divided into two temporal groups: group hospitalized between 2015 and 2020 (*n* = 82) and group hospitalized between 2021 and 2023 (*n* = 78). Demographic data, length of hospitalization, disease severity, renal involvement, and treatment were compared between the groups. **Results**: Patient age did not differ significantly between the groups (7.2 ± 3.82 vs. 6.8 ± 3.55 years; *p* = 0.469). The mean length of hospitalization decreased from 10.2 ± 4.78 to 7.9 ± 3.18 days (*p* < 0.001). The proportion of patients classified as having severe IgAV was lower in the 2021–2023 group than in the 2015–2020 group (9.0% vs. 20.7%; *p* = 0.046). Urinary abnormalities during hospitalization were observed in 16.7% of patients in the 2021–2023 group and in 40.2% of patients in the 2015–2020 group (*p* < 0.001). After the onset of the pandemic, antibiotics and etamsylate were used significantly less frequently, whereas the frequency of glucocorticoid treatment did not change significantly. **Conclusions**: Compared with the 2015–2020 period, children hospitalized with IgAV in 2021–2023 had shorter hospital stays, lower CRP concentrations, and less frequent urinary abnormalities, while antibiotics and etamsylate were used less frequently. The proportion of patients classified as having severe disease was also lower, although this difference was attenuated in sensitivity analysis. These temporal differences may reflect changes in infection epidemiology, clinical practice, and healthcare organization after the onset of the COVID-19 pandemic. However, the available data do not allow a direct relationship with SARS-CoV-2 infection to be established.

## 1. Introduction

Immunoglobulin A vasculitis (IgAV; formerly Henoch–Schönlein purpura; HSP [1,2]) is a systemic small-vessel vasculitis whose principal histopathological feature is the deposition of IgA1-containing immune complexes in affected tissues. It is an autoimmune disorder occurring predominantly in the paediatric population, with an incidence of 3–26.7 cases per 100,000 children. The disease is considerably less common in adults, with an annual incidence of 0.8–1.8 cases per 100,000 people [3,4,5].

The aetiology of IgAV is complex and involves both genetic susceptibility and environmental factors [6,7]. Antigenic stimulation is considered an important initiating factor. The pathogenesis involves the abnormal glycosylation of IgA1, formation of immune complexes, activation of the complement system, and ultimately vascular endothelial injury [8,9,10]. In many patients, disease onset is preceded by an infection, most commonly an upper respiratory tract infection [11].

The clinical presentation of IgAV is heterogeneous. The essential and mandatory classification criterion is purpura or petechiae unrelated to thrombocytopenia, typically with predominant involvement of the lower extremities. Other manifestations include arthralgia or arthritis, gastrointestinal symptoms, and renal involvement. In most children, the disease is mild and self-limiting. However, some patients develop severe organ complications, particularly involving the kidneys and gastrointestinal tract [1,7,12,13,14,15,16].

Renal involvement is of major prognostic importance [15,16,17]. Renal manifestations may occur during the acute phase of the disease or develop later. Therefore, continued follow-up is required after resolution of the skin lesions. The most recent nephrology recommendations emphasize repeated urinalysis, quantitative assessment of proteinuria, blood pressure measurement, and evaluation of renal function in children with IgAV [17].

The COVID-19 pandemic substantially changed the epidemiology of infections in paediatric patients. Social distancing, school and kindergarten closures, isolation of infected individuals and increased emphasis on the use of disinfectants reduced transmission of many respiratory pathogens, including SARS-CoV-2 [18,19,20]. During the early phases of the pandemic, a marked decline in the incidence of respiratory syncytial virus (RSV) and influenza infections was observed, together with a shift in their typical seasonal patterns [18,21]. Following the gradual lifting of restrictions, infection rates increased again, with outbreaks occurring outside the usual seasonal periods and with changes in the age distribution of affected children. At the same time, the pandemic affected the organization of healthcare. In many centres elective hospital admissions and outpatient visits were reduced, telemedicine consultations were introduced and hospital admission criteria became more restrictive in order to reduce the risk of infection transmission. In addition, molecular diagnostics for viral infections became more widely available. These changes may have influenced both the duration of hospitalization and the profile of patients referred for hospital treatment. Therapeutic practice also evolved, including a more selective approach to antibiotic use and greater emphasis on symptomatic treatment of viral infections [18,19,20,21].

The current literature contains only a limited number of recent studies evaluating the impact of the COVID-19 pandemic on the clinical course of IgAV in paediatric populations in European countries.

The aim of this study was to compare the demographic profile, length of hospitalization, disease severity, frequency of renal involvement and treatment patterns in children hospitalized with IgAV before and after the onset of the COVID-19 pandemic, in central Poland.

## 2. Materials and Methods

### Study Design and Patients

A retrospective, multicentre observational study was conducted based on the analysis of medical records of patients hospitalized between 2015 and 2023 at three tertiary paediatric referral centres in central Poland, representing general paediatric, paediatric nephrology and paediatric rheumatology. Patients were admitted through different referral pathways: some presented directly to the emergency department or hospital admission unit, whereas others were referred for hospitalization after an initial assessment by a primary care physician.

A total of 160 patients were included in the analysis. Potentially eligible children aged 0–18 years were identified on the basis of the International Classification of Diseases, 10th Revision (ICD-10) diagnostic codes: M31.0 (autoimmune vasculitis) and D69.0 (allergic purpura). Medical records identified using these codes were individually reviewed to confirm eligibility and the diagnosis of IgAV. Patients were eligible if IgAV was suspected either before admission or during hospitalization for another condition. The diagnosis was based on the clinical presentation and the EULAR/PRINTO/PRES classification criteria [14]. The presence of purpura or petechiae was required, together with at least one of the following: abdominal pain, arthralgia/arthritis, renal involvement, or characteristic histopathological findings. Patients aged > 18 years and those diagnosed with IgAV outside the 2015–2023 study period were excluded.

Patients were divided into two temporal groups: a group hospitalized between 2015 and 2020 (*n* = 82) and a group hospitalized between 2021 and 2023 (*n* = 78). The year 2021 was chosen as the cut-off because it represented the first full calendar year after the onset of the COVID-19 pandemic, during which sustained pandemic-related changes in paediatric healthcare organization and infection epidemiology were already established. Accordingly, all patients hospitalized in 2020 were classified in the earlier study period, regardless of the exact timing of the introduction of local pandemic restrictions. As 2020 represented a heterogeneous transitional year, a sensitivity analysis excluding patients hospitalized in 2020 was performed to assess the robustness of the main findings. The main outcomes assessed in the between-period differences were length of hospitalization, urinary abnormalities during hospitalization, exploratory disease severity, and treatment patterns. Additional analyses included demographic characteristics, inflammatory markers, potential disease triggers, and the occurrence of IgAVN.

The analysis included the course of hospitalization, including length of stay, clinical manifestations, organ involvement, laboratory parameters and treatment, with particular emphasis on glucocorticoids, antibiotics, and etamsylate. The age structure of the study population, potential disease triggers, frequency of urinary abnormalities and IgA vasculitis nephritis (IgAVN), and selected factors associated with a more severe course of IgAV were also assessed. Laboratory parameters were based on the first available measurements obtained during the hospitalization. Urinalysis was routinely performed during hospitalization in both study periods as part of the standard assessment of children with suspected IgAV. For the purposes of the comparative analysis, urinary abnormalities during hospitalization were defined as the presence of haematuria and/or proteinuria detected on urinalysis. IgAVN was analysed separately and was defined as persistent urinary abnormalities (including haematuria and/or proteinuria), accompanied by impaired renal function and confirmed by paediatric nephrology assessment at a referral centre. Because of the retrospective nature of the study, complete information regarding kidney biopsy was not available for all patients with IgAVN; therefore, histological confirmation was not required for inclusion in the IgAVN subgroup. Information on potential triggering factors, including previous infections, medications and vaccinations, was extracted from the available medical records. However, COVID-19 vaccination status was not systematically documented for all patients and therefore could not be included as a separate variable in the comparative analysis. Missing data were not imputed and analyses were based on the available data for each variable.

Disease severity was additionally classified using a study-specific, non-validated exploratory composite IgAV severity score. The scale was based on clinical data and the EULAR/PRINTO/PRES [14] and IPNA [17] recommendations. Hospitalized patients were assessed according to the observed clinical features (Table 1).

To minimize potential bias related to treatment decisions and healthcare organization, a sensitivity analysis was performed using a modified severity score excluding glucocorticoid treatment and length of hospitalization. To assess the robustness of the classification, alternative thresholds for severe disease were also examined.

Statistical analysis was performed to characterize the clinical features of the patients and assess differences between the study groups, with particular emphasis on IgAV severity, treatment and urinary abnormalities. The distribution of continuous variables was assessed using the Shapiro–Wilk test, and data were presented as medians or means with standard deviations, as appropriate. Categorical variables were presented as counts or percentages. Comparisons were performed using the nonparametric Kruskal–Wallis test, the Mann–Whitney U test, or Student’s *t*-test, as appropriate. Categorical variables were analyzed using Pearson’s chi-square test, the chi-square test with Yates’ correction, Fisher’s exact test, or permutation tests with the Monte Carlo method. A *p* value < 0.05 was considered statistically significant. Calculations were performed using Statistica 13 (StatSoft Inc., Tulsa, OK, USA); selected permutation analyses were additionally performed in Python 3.15.2 using the NumPy 2.5.4 and SciPy 1.18.1 packages.

The study was approved by the Bioethics Committee of the Polish Mother’s Memorial Hospital Research Institute in Lodz (Opinion No. 49/2024 of 14 May 2024). Approval was obtained from the hospitals responsible for patient care to use anonymized patient data.

## 3. Results

The analysis included 160 children and adolescents, including 76 girls (47.5%) and 84 boys (52.5%), diagnosed with IgAV and hospitalized between 2015 and 2023. The group hospitalized before the onset of the COVID-19 pandemic, covering the years 2015–2020, comprised 82 patients, whereas 78 patients were included in the group hospitalized between 2021–2023. In the analysed population, two cases of IgAV were preceded by confirmed SARS-CoV-2 infection. The interval between SARS-CoV-2 infection and the onset of IgAV manifestations was approximately 6 weeks and 2 weeks, respectively.

There was no significant difference in age between the two groups. The mean age of patients was 7.2 ± 3.82 years before the pandemic and 6.8 ± 3.55 years after the onset of the pandemic (*p* = 0.469), indicating a similar age profile in the two periods.

A significant difference was observed in the length of hospitalization. The mean hospital stay was 10.2 ± 4.78 days in the earlier period and 7.9 ± 3.18 days in the later period (*p* < 0.001), corresponding to a reduction of 2.3 days in the mean length of hospitalization in the 2021–2023 period (Table 2).

Analysis of IgAV severity using the study-specific exploratory severity score demonstrated a significantly lower proportion of patients with severe disease in the 2021–2023 group. Severe disease was observed in 17 of 82 patients (20.7%) in the earlier group and in 7 of 78 patients (9.0%) in the later period (*p* = 0.046).

In the sensitivity analysis excluding glucocorticoid treatment and length of hospitalization from the severity score, severe IgAV was identified in 7 of 82 patients (8.5%) in the 2015–2020 period and in 1 of 78 patients (1.3%) in the 2021–2023 period. The difference was attenuated and did not reach statistical significance (*p* = 0.064), although the direction of the association remained unchanged. The same pattern, with a higher frequency of severe IgAV in the earlier period, was preserved across alternative severe-disease thresholds of ≥7 to ≥9 points. The results of the sensitivity analysis are presented in Supplementary Table S1.

Laboratory parameters reflecting the intensity of the inflammatory response were also compared. The median CRP concentration in the 2015–2020 period was 6.05 mg/L (0.1–108.0 mg/L), whereas in 2021–2023 it was significantly lower at 1.15 mg/L (0.2–38.5 mg/L; *p* < 0.001). Significant differences were also observed in urinary abnormalities detected during hospitalization. In the 2015–2020 group, haematuria and/or proteinuria were observed in 33 patients (40.2%), compared with 13 patients (16.7%) in the 2021–2023 group (*p* < 0.001) (Table 3).

Among patients with urinary abnormalities, isolated haematuria was observed in 10 patients, proteinuria in 5 patients, and both changes in 31 patients. Hypertension was present in 2 patients, whereas impaired renal function was observed in 13 children.

In addition, IgA vasculitis nephritis (IgAVN), defined as persistent urinary abnormalities (including haematuria and/or proteinuria), accompanied by impaired renal function and confirmed by paediatric nephrologist, was diagnosed in 13 of 160 patients (8.1%) in the overall cohort. Patients with IgAVN were significantly older, with a mean age of 9.4 ± 3.33 years, compared with 6.5 ± 3.48 years in patients without nephritis (*p* = 0.005). No significant differences were found between patients with and without nephropathy in serum IgA (*p* = 0.094), CRP (*p* = 0.30), or D-dimer levels (*p* = 0.850). Analysis of potential triggers showed a significant association between urinary tract infection and the development of nephropathy. A urinary tract infection preceding IgAV was identified in 7.7% of patients with IgAVN and in 0.7% of patients without nephropathy (*p* = 0.029).

Further analysis evaluated differences in treatment patterns. Following the onset of the pandemic, antibiotic therapy was used significantly less frequently. Antibiotics were administered to 57 of 82 patients (69.5%) hospitalized between 2015 and 2020 and to 32 of 78 patients (41.0%) hospitalized between 2021 and 2023 (*p* < 0.001). Haemostatic agents such as etamsylate were also used significantly less frequently in the later period, in 70 patients (85.4%) and 52 patients (66.7%) respectively (*p* = 0.006).

There was no significant difference in the frequency of glucocorticoid use. Glucocorticoid treatment was administered to 25 patients (30.5%) in the 2015–2020 group and 18 patients (23.1%) in the 2021–2023 group (*p* = 0.291) (Table 4).

In a sensitivity analysis excluding patients hospitalized in 2020, the direction and main between-period differences remained unchanged, supporting the robustness of the primary findings.

In summary, the 2021–2023 period was characterized by a shorter hospital stay, a lower proportion of severe IgAV, less frequent urinary abnormalities during hospitalization, lower CRP values, and reduced use of antibiotics and etamsylate, with no significant change in the frequency of glucocorticoid treatment.

**Figure 1:**
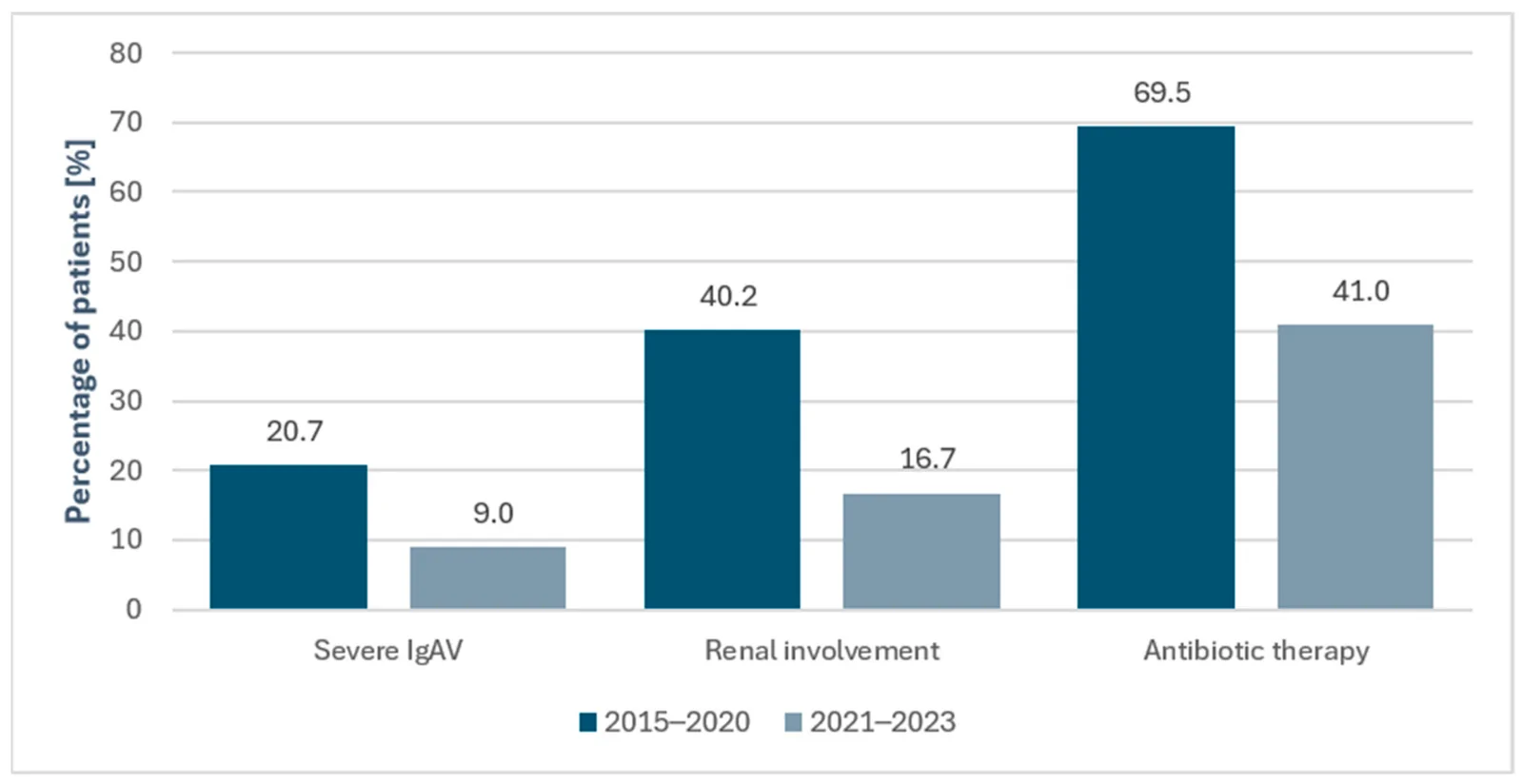
Significant differences between the 2015–2020 and 2021–2023 study periods.

Significant differences in the analysed parameters are presented in Figure 1.

## 4. Discussion

In this multicentre retrospective cohort, children hospitalized with IgAV in 2021–2023 had shorter hospital stays, lower CRP concentrations, and less frequent renal involvement than those hospitalized in the earlier period. The proportion of patients classified as having severe IgAV was also lower in the later period. Importantly, the between-period difference in disease severity was attenuated after exclusion of hospitalization duration and glucocorticoid treatment from the exploratory score, although the direction of the effect remained unchanged, suggesting that healthcare and treatment-related components contributed partly to the original classification.

Antibiotics and etamsylate were also used less frequently, whereas glucocorticoid use remained unchanged. Patient age at diagnosis was comparable between the two periods. Overall, these findings suggest that both the clinical profile of hospitalized children with IgAV and therapeutic practice changed after the onset of the COVID-19 pandemic. However, the retrospective design does not allow these differences to be attributed directly to SARS-CoV-2 infection or pandemic-related restrictions.

Most of the available data on the association between COVID-19 and IgAV are derived from case reports, case series, and literature reviews. Farooq et al. [18], in a systematic review, identified 13 cases of IgAV or IgAVN associated with COVID-19 and 4 cases of IgAVN following COVID-19 vaccination. The authors suggested that SARS-CoV-2 infection may represent a potential trigger initiating an immunological cascade leading to the development of IgAV, particularly in genetically predisposed individuals. Similar observations were reported by Nikolaishvili et al. [11], Messova et al. [19], and Suszek et al. [20]; however, these authors emphasized that the role of SARS-CoV-2 as a trigger of IgAV in both adults and children remains uncertain and requires further investigation.

In the paediatric population, the case report by Machura et al. [22], published in 2023, is also relevant. It described a 7-year-old child who developed IgAV after SARS-CoV-2 infection and suggested a possible triggering role of the infection.

Several immunological mechanisms may explain a potential relationship between SARS-CoV-2 infection and IgAV. SARS-CoV-2 induces mucosal and systemic immune responses, including IgA production, cytokine release, complement activation, and endothelial inflammation, which may promote IgA immune-complex formation and vascular deposition in susceptible individuals. More severe COVID-19 could theoretically enhance these mechanisms through greater systemic inflammation and endothelial injury. However, a direct relationship between the severity of COVID-19 and subsequent IgAV severity has not been established.

In the present cohort from the Lodz region, only two patients had documented SARS-CoV-2 infection preceding IgAV, and the severity of the infection was not systematically recorded. This observation is consistent with the possibility that SARS-CoV-2 may act as one of several infectious triggers, although the small number does not allow any conclusions regarding causality.

COVID-19 vaccination should also be considered as a potential immunological trigger. Case reports and systematic reviews have described new-onset vasculitis following SARS-CoV-2 vaccination, including IgAV, although such events appear to be rare and a causal relationship has not been established. A recent systematic review of vaccine-associated vasculitis identified 158 reported cases of vasculitis following COVID-19 vaccination, including 24 cases of IgAV [23].

In the present cohort, vaccination-related IgAV was identified in only one patient (0.6%); however, this case was associated with routine childhood immunization rather than COVID-19 vaccination. COVID-19 vaccination status was not systematically available in medical records, and therefore its potential contribution to differences between the study periods could not be assessed. Consequently, a possible modifying effect of COVID-19 vaccination on the clinical phenotype observed in the later study period cannot be excluded.

Epidemiological studies provide important additional insight into the broader impact of the pandemic on the clinical course of IgAV [24,25]. A French nationwide study including 1988 patients demonstrated a reduction in the incidence of IgAV among children during the COVID-19 pandemic. The authors postulated that this observation may have resulted from reduced exposure to respiratory tract infections, which are recognized as one of the main factors preceding IgAV [25]. In addition, a population-based study by Felix et al. [26], including 9790 children hospitalized with IgAV between 2015–2023, showed a marked decline in IgAV cases during the COVID-19 restriction period, followed by an increase after the restrictions were lifted. These changes were temporally associated with the circulation of common seasonal pathogens, particularly *Streptococcus pneumoniae*, *Streptococcus pyogenes*, and rhino- and enteroviruses, whereas no clear association with SARS-CoV-2 infections was demonstrated.

These findings raise the possibility that the impact of the pandemic on IgAV may also be indirect, resulting not only from SARS-CoV-2 infection itself but also from changes in the epidemiology of other pathogens. This appears particularly relevant in IgAV, as disease onset is frequently preceded by infection, especially of the respiratory tract.

Key data are also provided by the study published in 2026 by Wang et al. [27], which included 1469 children with IgAV hospitalized before the pandemic and in the post-pandemic period. The authors reported an increased proportion of hospitalizations due to IgAV, a higher frequency of renal involvement, more frequent progression to IgAVN and a shift in the clinical presentation toward more severe-disease phenotypes after the COVID-19 pandemic. The authors also observed a significant increase in *Mycoplasma pneumoniae* infections, from 15.8% to 34.0%, and suggested that the altered profile of respiratory pathogens after the pandemic, particularly the higher prevalence of *M. pneumoniae* infections, may have promoted excessive immune activation.

Similar observations regarding renal involvement were reported by Batu et al. [28] in an international, multicenter study of children with vasculitis temporally associated with exposure to SARS-CoV-2. Among 30 patients with COVID-19-associated IgAV, renal involvement was observed in 43.3% of children, compared with 17.6% in the pre-pandemic control group (*p* = 0.002). These findings may suggest that, in a subset of patients, IgAV developing in temporal association with SARS-CoV-2 infection may present with a more severe phenotype, particularly with respect to renal involvement.

These observations differ from the findings of the present study. In the Lodz cohort, comparison of both groups showed no significant differences in patient age at diagnosis, suggesting that the demographic profile remained similar across the two periods. Significant differences were, however, observed in the length of hospitalization: after the onset of the pandemic, the mean hospital stay was significantly shorter (7.9 vs. 10.2 days; *p* < 0.001). In addition, the 2021–2023 period was associated with a significantly lower proportion of patients classified as having severe IgAV and a lower frequency of urinary abnormalities during hospitalization. These observations were accompanied by significantly lower CRP values after the pandemic, indicating lower inflammatory activity among hospitalized patients. The lower frequency of renal involvement was assessed on the basis of urinalysis abnormalities. Several factors may account for these differences. The study by Wang et al. [27] covered the years 2023–2025, representing a later post-pandemic phase than the 2021–2023 period analysed in the present cohort. In addition, different definitions of severe disease and renal involvement may have also been applied. Methodological differences are also relevant: Batu et al. [28] analysed a selected group of patients with documented exposure to SARS-CoV-2 within the three months preceding the development of IgAV, whereas the Polish study compared the entire database of children diagnosed with IgAV without stratification according to the triggering factor. Furthermore, disease severity in the present cohort was assessed using a study-specific, non-validated composite severity score. These factors may partly explain the divergent findings reported across studies and highlight the need for cautious interpretation of the potential impact of SARS-CoV-2 infection on the development of IgAV and IgAVN.

Several factors may explain the apparently milder clinical phenotype observed in the 2021–2023 period. Changes in the circulation and spectrum of infectious triggers after the onset of the COVID-19 pandemic may have resulted in differences in the magnitude of systemic inflammatory responses, which is consistent with the lower CRP concentrations and less frequent urinary abnormalities observed in the later period. At the same time, pandemic-related changes in referral pathways, hospital admission criteria, and clinical practice may have altered the profile of children admitted to tertiary centres. Moreover, the attenuation of the between-period difference in disease severity after exclusion of glucocorticoid treatment and hospitalization duration from the exploratory score indicates that healthcare- and treatment-related factors contributed partly to the original severity classification. Thus, the lower observed severity in the later period is likely to reflect a combination of epidemiological, healthcare-related, and methodological factors rather than a direct effect of SARS-CoV-2 infection.

The importance of age and laboratory parameters in the assessment of renal risk is also supported by more recent studies. In 2025, Shen et al. [29] analysed data from 384 children with IgAV hospitalized between 2020 and 2023 and found that older age, lower serum albumin, and higher total cholesterol were independent factors associated with the development of IgAVN. Their observations regarding age are consistent with those from the Lodz cohort and indicate a higher frequency of IgAVN among older children.

Another noteworthy finding was the shorter duration of hospitalization in the later period. A similar tendency was demonstrated in another study from Lodz analysing the epidemiology of RSV infections between 2016 and 2024, in which changes in seasonality, patient age, disease severity and hospitalization patterns were reported [21]. It was attributed to the reorganization of healthcare and shifts in the immunological profile of children following the pandemic. The similarity between epidemiological changes observed in RSV infection and IgAV may support the hypothesis that the COVID-19 pandemic affected the functioning of the healthcare system as a whole rather than only the dynamics of specific diseases. Comparable pandemic-related observations have also been reported in dermatology. Both acute COVID-19 and post-COVID conditions have been associated with a broad spectrum of cutaneous manifestations, including inflammatory and immune-mediated lesions, while the pandemic also altered patterns of healthcare utilization in dermatological practice [30,31,32]. Taken together, these findings provide additional context for the broader systemic and healthcare-related effects of the COVID-19 pandemic across different clinical specialties.

Changes in therapeutic practice were also observed. Antibiotics and etamsylate were used less frequently in the later study period, whereas the frequency of glucocorticoid use remained unchanged. This may reflect an evolution toward more selective treatment strategies based on disease severity and organ involvement. Current recommendations do not support the routine use of glucocorticoids for the prevention of IgAVN, although they remain indicated in selected patients with severe extrarenal manifestations or clinically significant renal involvement [17,33]. Antibiotics were not administered as treatment for IgAV itself, but were used when clinically indicated, based on the patient’s clinical condition, elevated inflammatory markers and, when available, microbiological confirmation of bacterial infection. Because detailed indications for antibiotic treatment were not consistently documented in the retrospective records, the observed reduction in antibiotic use should be interpreted primarily as a temporal change in treatment practice rather than as direct evidence of improved antimicrobial stewardship. Etamsylate was used in the participating centres as symptomatic haemostatic treatment according to local clinical practice rather than as a guideline-recommended disease-modifying therapy for IgAV. Therefore, the reduction in its use should likewise be interpreted as a change in local therapeutic practice over time.

Early identification of children at risk of renal involvement during the course of IgAV remains an important clinical challenge. In a systematic review published in 2025, Liao et al. [34] found that most prediction models for the development of IgAV nephropathy were at high risk of bias and that most had not undergone external validation. This highlights the continuing need for further prospective studies on validated tools for prediction of renal complications.

Altogether, the available evidence suggests that the impact of the COVID-19 pandemic on IgAV is likely multifactorial. SARS-CoV-2 may act as a direct infectious trigger in selected patients, while broader pandemic-related changes in the circulation of seasonal pathogens, healthcare organization, diagnostic pathways and therapeutic practice may also influence the observed clinical phenotype of hospitalized children with IgAV. Importantly, the present study was designed primarily as a comparison of two historical periods rather than as an analysis of the direct effects of individual SARS-CoV-2 infection or COVID-19 vaccination on IgAV.

### Strengths and Limitations

Several strengths of the study are worth highlighting. Its multicentre design included three large paediatric centres representing different profiles of care, including general paediatrics, nephrology and rheumatology, which increases the clinical heterogeneity and relevance of the study population. The relatively long observation period allowed for the assessment of changes occurring before and after the onset of the COVID-19 pandemic. In addition, the study provides data from a Central European paediatric population, which remains underrepresented in the literature. Another strong point is the simultaneous assessment of both clinical characteristics and changes in therapeutic practice.

A number of limitations should also be acknowledged. The retrospective design may have affected the completeness and uniformity of the available clinical data and precludes establishing causal relationships between the COVID-19 pandemic and the observed changes in IgAV presentations. Detailed data on changes in hospital admission criteria, referral pathways and discharge policies were not available, therefore their potential impact on the observed differences can only be inferred. Residual confounding related to age, sex, recruiting centres, and temporal changes in clinical practice cannot be excluded. The unequal duration of the two study periods may also have influenced the observed temporal differences. Disease severity was assessed using a study-specific, non-validated composite severity score, which limits comparability with other cohorts. Although sensitivity analysis excluding healthcare-related factors, such as treatment- and hospitalization-related components, showed a similar direction of effect, the difference was attenuated and no longer statistically significant, underscoring the exploratory nature of the severity score. In addition, COVID-19 vaccination status was not systematically available, preventing assessment of its potential influence on the subsequent clinical phenotype of IgAV. Classifying 2020 within the earlier study period is another potential limitation because it was a heterogeneous transitional year. However, sensitivity analysis excluding 2020 showed a similar pattern of findings. Finally, renal involvement may have been underestimated because systematic long-term follow-up after hospital discharge was not available and delayed manifestations of IgAVN could therefore have been missed.

## 5. Conclusions

Children hospitalized with IgAV in 2021–2023 had shorter hospital stays, lower CRP concentrations, and less frequent urinary abnormalities than those hospitalized in the 2015–2020 period. Therapeutic practice also changed, with less frequent use of antibiotics and haemostatic agents. These temporal differences may reflect changes in infection epidemiology, regional variation in the clinical phenotype of vasculitis, clinical practice, and healthcare organization after the onset of the COVID-19 pandemic. Given the retrospective design, a direct causal relationship with SARS-CoV-2 infection cannot be established.

## Figures and Tables

**Table 1:** Study-specific, non-validated exploratory composite IgAV severity score.

Category		Criteria	Points
**1.** **Glucocorticoid treatment**		NO	0
		YES	1
**2.** **Length of hospitalization**		0–7 days	0
		7–14 days	1
		>14 days	2
**3.** **Inflammatory response**	CRP * **(*N* < 10 mg/L)**	<10 mg/L	0
		10–50 mg/L	1
		>50 mg/L	2
	**D-dimer** **(*N* < 500 ng/mL)**	<500 ng/mL	0
		500–1000 ng/mL	1
		>1000 ng/mL	2
**4.** **Number of organs involved** **(excluding skin)**		Arthralgia/abdominal pain	1
		Gastrointestinal tract (faecal occult blood)	1
		Joints (ultrasound changes ex. effusion)	1
		Kidneys (urinalysis abnormalities)	1
		Nephropathy manifestations (significant proteinuria, >0.5 g/day)	2
		Other organs (scrotum, lungs, CNS *)	2

* CRP—C-reactive protein; CNS—central nervous system. TOTAL (maximum 15 points): Mild course: 0–4 points; Moderate course: 5–8 points; Severe course: 9–15 points. Based on the total score, disease course was classified as mild (0–4 points), moderate (5–8 points), or severe (9–15 points).

**Table 2:** Characteristics of patients in the 2015–2020 and 2021–2023 study periods.

Parameter (Mean ± SD)	2015–2020 (*N* = 82)	2021–2023 (*N* = 78)	*p*-Value	Statistical Test
Age (years)	7.2 ± 3.82	6.8 ± 3.55	0.469	Student’s *t*-test
Length of hospitalization (days)	10.2 ± 4.78	7.9 ± 3.18	<0.001	Welch’s *t*-test

**Table 3:** Comparison of disease severity and urinary abnormalities during hospitalization in the 2015–2020 and 2021–2023 study periods.

Parameter	2015–2020 (*N* = 82)		2021–2023 (*N* = 78)		*p*-Value	Statistical Test
	Number of Patients [*n*]	Percentage [%]	Number of Patients [*n*]	Percentage [%]		
Severe IgAV	17	20.7	7	9.0	0.046	Fisher’s test
Urinary abnormalities	33	40.2	13	16.7	<0.001	Chi-square test

**Table 4:** Comparison of therapeutic characteristics between the 2015–2020 and 2021–2023 study periods.

Treatment	2015–2020 (*N* = 82)		2021–2023 (*N* = 78)		*p*-Value	Statistical Test
	Number of Patients [*n*]	Percentage [%]	Number of Patients [*n*]	Percentage [%]		
Etamsylate	70	85.4	52	66.7	0.006	Chi-square test
Antibiotics	57	69.5	32	41.0	<0.001	Chi-square test
Glucocorticoids	25	30.5	18	23.1	0.291	Chi-square test

## Data Availability

Data are available from the corresponding author upon reasonable request. Due to legal and ethical constraints under Polish law and GDPR, the dataset, which includes anonymized data from minors, cannot be made publicly available. Access may be granted under data use agreements ensuring compliance with applicable regulations.

## Supplementary Materials

The following supporting information can be downloaded at: https://www.mdpi.com/article/10.3390/jcm15218399/s1; Table S1: Sensitivity analysis of severe IgAV classification after exclusion of hospitalization duration and glucocorticoid treatment from the severity score.

## Abbreviations

The following abbreviations are used in this manuscript:

IgAV	Immunoglobulin A vasculitis
IgAVN	IgA vasculitis nephritis
EULAR/PRINTO/PRES	European Alliance of Associations for Rheumatology/Paediatric Rheumatology International Trials Organisation/Paediatric Rheumatology European Society
IPNA	International Pediatric Nephrology Association
