# Supplementary material for: Did the COVID-19 Pandemic Change the Clinical Presentation and Management of Paediatric IgA Vasculitis? A Multicentre Retrospective Cohort Study
Source: J Clin Med. 2026 Oct 28;15(21):8399. doi: 10.3390/jcm15218399 (PMC13763744; doi:10.3390/jcm15218399)
Supplement: Supplementary file 1 [file jcm-15-08399-s001.zip › jcm-4624880-supplementary.pdf]

Supplementary Table S1. Sensitivity analysis of severe IgAV classification after exclusion of hospitalization duration and glucocorticoid treatment from the severity score.

Definition of severe IgAV	2015-2020 (N = 82)	2021-2023 (N = 78)	Fisher's exact test, p-value
Original severity score	17 (20.7%)	7 (9.0%)	0.046
Sensitivity-analysis score (excluding hospitalization duration and glucocorticoid use)	7 (8.5%)	1 (1.3%)	0.064
